# Rapidly progressive dementia in a middle-aged woman: A case of probable sporadic Creutzfeldt-Jakob disease

**DOI:** 10.3892/mi.2025.288

**Published:** 2025-12-01

**Authors:** Veramar R. Jarquín-Rios, Whiny S. Rodríguez-Gutierrez, Eduardo Daberkow-Sánchez, David A. Flores-Garcia, Alina Teresa Sánchez Vázquez

**Affiliations:** Department of Internal Medicine, Hospital General Dr. Belisario Domínguez ISSSTE, Tuxtla Gutiérrez, Chiapas 29040, Mexico

**Keywords:** Creutzfeldt-Jakob disease, prion disease, myoclonus, rapidly progressive dementia, 14-3-3 protein

## Abstract

Creutzfeldt-Jakob Disease (CJD) is a rare, rapidly progressive and fatal prion encephalopathy characterized by cognitive deterioration, motor dysfunction and myoclonus. The present study describes the case of a 55-year-old female patient with a history of controlled hypertension who presented with subacute cognitive decline, memory impairment, executive dysfunction and behavioral changes. Within weeks, her condition worsened with gait instability, upper limb dysmetria, ataxia and myoclonus, progressing to global dementia. An electroencephalography revealed periodic triphasic complexes at 1-2 Hz, brain magnetic resonance imaging revealed bilateral parieto-occipital cortical hyperintensities, and cerebrospinal fluid (CSF) analysis was positive for 14-3-3 protein, supporting the diagnosis of sporadic CJD (sCJD). The present case report illustrates a classical presentation of sCJD with a rapidly evolving clinical course. The integration of clinical features with electroencephalographic, neuroimaging and CSF biomarkers enabled a strong presumptive diagnosis. Despite the absence of curative treatment, early recognition remains essential to guide supportive management and avoid unnecessary interventions. Differential diagnosis with entities, such as autoimmune encephalitis and metabolic encephalopathies should be considered. In the case described herein, collaboration between psychiatry, neurology and palliative care ensured timely diagnosis and support.

## Introduction

Creutzfeldt-Jakob disease (CJD) is a rare, fatal and rapidly progressive neurodegenerative disorder caused by misfolded prion proteins (PrP^Sc^), which induce abnormal conformational changes in normal cellular prions (PrP^C^), leading to widespread neuronal loss, spongiform changes, and gliosis (1,2). The sporadic form [sporadic CJD (sCJD)] accounts for 85-90% of cases, with an estimated annual incidence of 1-1.5 per million in the population worldwide (3-5) sCJD primarily affects individuals aged 50-70 years and is characterized by rapidly progressive dementia, myoclonus, ataxia, visual disturbances and pyramidal or extrapyramidal signs (1,5).

As sCJD progresses rapidly and shares features with autoimmune, infectious, metabolic, toxic and neoplastic disorders, timely recognition is critical for accurate diagnosis and appropriate management. Probable sCJD is diagnosed through a combination of clinical findings and supportive paraclinical evidence, including periodic sharp wave complexes on an electroencephalography (EEG), cortical ribboning or basal ganglia hyperintensities on magnetic resonance imaging (MRI) (6-8), and positive cerebrospinal fluid (CSF) biomarkers, such as 14-3-3 protein or real-time quaking-induced conversion (RT-QuIC) assays (9).

The present study describes the case of a 55-year-old female patient whose initial psychiatric symptoms rapidly progressed to severe cognitive and motor decline, ultimately meeting criteria for probable sCJD. By highlighting the integration of clinical assessment, neuroimaging, EEG and CSF biomarkers, the present case report aimed to emphasize the diagnostic challenges and the importance of early multidisciplinary evaluation in rapidly progressive neurocognitive syndromes.

## Case report

### Presentation of the case

A 55-year-old woman with a history of well-controlled systemic arterial hypertension under regular treatment, and no prior neurological or psychiatric conditions, presented to the Department of Internal Medicine, Hospital General Dr. Belisario Domínguez ISSSTE, Tuxtla Gutiérrez, Chiapas, Mexico, in February 4, 2025, with an 8-week history of gradually progressive cognitive decline. The initial symptoms included short-term memory loss, impaired executive functions and behavioral alterations, such as irritability, mild disinhibition and apathy, leading to an initial psychiatric evaluation under the suspicion of major depressive disorder.

Her condition rapidly deteriorated, progressing to gait instability with frequent falls, wide-based ataxia, dysarthria of the scanning type and spontaneous myoclonus, which became multifocal and stimulus-sensitive, predominantly affecting the left side. The cognitive deterioration advanced to overt dementia with temporal-spatial disorientation, partial mutism, and severe functional impairment.

Upon admission, her Glasgow coma scale score was 13/15. She was alert but minimally verbal, with axial rigidity, bradykinesia, bradyphrenia and hypokinetic dysarthria. Global motor responses to painful stimuli were preserved, without lateralization. Cerebellar signs, including dysmetria and limb ataxia, were evident, as well as multifocal myoclonus triggered by external stimuli. No focal motor or sensory deficits were observed.

**Figure 1:**
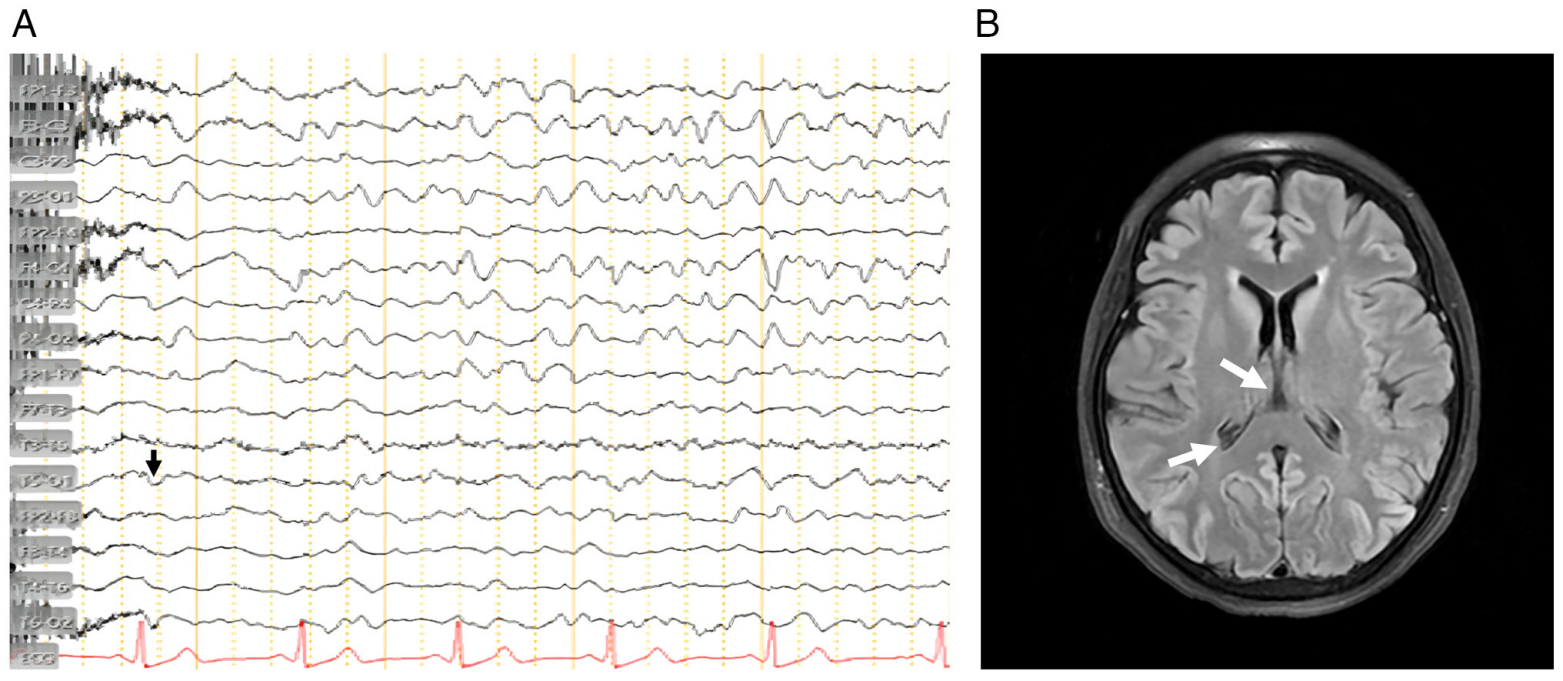
(A) Electroencephalogram from the patient illustrating generalized periodic triphasic complexes at 1-2 Hz, a hallmark pattern consistent with prion disease. (B) Axial brain magnetic resonance imaging in FLAIR sequence demonstrating bilateral hyperintensities in the parieto-occipital cortex and basal ganglia (cortical ribboning sign), corresponding to the findings of the patient. Arrows indicate areas of cortical ribboning and basal ganglia involvement. Only the sequences displayed are shown; no gadolinium contrast images were included. Arrows indicate areas of cortical and basal ganglia involvement.

An EEG demonstrated generalized periodic discharges with triphasic complexes at 1-2 Hz, a pattern typical of prion encephalopathies. The EEG findings are illustrated in Fig. 1A. A brain MRI in FLAIR and DWI sequences revealed cortical ribboning in the parieto-occipital cortex and basal ganglia, characteristic of CJD. Representative MRI images are shown in Fig. 1B.

CSF analysis was positive for 14-3-3 protein (50,026 AU/ml; reference, <20,000 AU/ml=negative), determined using an automated enzyme immunoassay (ELISA-based) method that reports results in arbitrary units (AU/ml) and are interpreted qualitatively (positive/negative) according to the manufacturer’s reference range. The test was performed using a monoclonal antibody specific for the 14-3-3 γ-isoform, analyzed with an automated chemiluminescence imaging system (Bio-Rad ChemiDoc MP; Bio-Rad Laboratories, Inc.). Routine CSF analyses revealed normal cell counts and biochemical parameters, and no evidence of infection or pleocytosis. A broad panel of paraneoplastic (anti-Hu, anti-Yo, anti-Ri, anti-Ma2, anti-CV2, amphiphysin), autoimmune (NMDA-R, AMPA-R, LGI1, CASPR2, GABA-B-R) and infectious (HSV, VZV, CMV, EBV, HIV, VDRL) panels were negative. Metabolic, including thyroid function, vitamin B12, folate, and ammonia, were within normal limits (10).

This clinical and diagnostic profile supported a probable diagnosis of sCJD. The case is notable for its initial psychiatric presentation and rapid neurological decline, highlighting the diagnostic challenges that may delay recognition of prion diseases in early stages.

### Diagnostic workup and imaging techniques

The diagnosis of probable sCJD was established according to the updated 2018 Centers for Disease Control and Prevention (CDC) diagnostic criteria and the UCSF Prion Center guidelines (2023), integrating the clinical course, characteristic EEG findings, MRI features and positive CSF biomarker (14-3-3 protein) (10).

### Diagnostic criteria applied

The diagnosis of probable sCJD was established based on the updated diagnostic criteria from the Centers for Disease Control and Prevention (CDC) and the UCSF Prion Center, which incorporate clinical features, EEG patterns, MRI findings, and CSF biomarkers (10,11). During hospitalization, the patient experienced progressive neurological deterioration, characterized by worsening cognitive decline, generalized myoclonus, and decreased level of consciousness. Subsequently, she developed healthcare-associated pneumonia complicated by acute respiratory distress syndrome (ARDS). Despite receiving supportive and palliative management, her condition continued to deteriorate, and she passed away on June 22, 2025.

### Ethical considerations

The present case report was conducted in accordance with the ethical standards of the institutional and national research committees, and with the 1964 Declaration of Helsinki and its later amendments (12). Written informed consent was obtained from the patient's legal representative for the publication of this case and any accompanying images. All data were anonymized to protect patient confidentiality. The case report was approved by the Health Research Coordination of the General Hospital ‘Dr. Belisario Domínguez’, ISSSTE, under official authorization no. CIS/0100/2025. The case did not involve experimental procedures and was based solely on routine clinical care.

## Discussion

sCJD is a subacute, fatal prion encephalopathy. Despite its low incidence rates, it requires a high index of suspicion in cases of rapidly progressive dementia, particularly in previously functional patients without a relevant neuropsychiatric history (2,13,14). The present case report exemplifies the diagnostic challenges posed by an early psychiatric presentation in a previously independent, middle-aged woman, in whom the rapid evolution of symptoms underscored the importance of close collaboration between psychiatry and neurology for timely recognition of neurodegenerative etiologies (3,15-19).

The progression of the condition of the patient from anterograde memory loss and executive dysfunction to ataxia, scanning dysarthria and multifocal myoclonus aligns with the characteristic triad of cognitive decline, myoclonus and cerebellar dysfunction described in cases of sCJD in the literature (6,7,20-22).

In the literature, an EEG demonstrated periodic triphasic complexes (observed in ~65-75% of cases), while an MRI revealed cortical ribboning in the parieto-occipital cortex and basal ganglia, a highly sensitive and specific imaging marker for prion disease. These findings, combined with a positive 14-3-3 protein assay in CSF, provided strong diagnostic support in the absence of histopathological confirmation (8,9,23,24) (Fig. 1B). The combination of EEG and MRI findings improved diagnostic confidence in the absence of histopathology (14,25,26).

The 14-3-3 protein result (50,026 AU/ml; reference, <20,000 AU/ml=negative) reflects a semi-quantitative positive reading from an enzyme immunoassay system. When interpreted alongside typical clinical and neuroimaging features, this biomarker significantly increases diagnostic confidence. Nevertheless, false positives may occur in acute neurological conditions, such as stroke or encephalitis; thus, the results should be interpreted within the clinical context (27-30). The 14-3-3 protein assay was performed using an automated enzyme immunoassay (ELISA-based) method that reports results in arbitrary units (AU/ml), which are interpreted qualitatively (positive/negative) according to the manufacturer's reference range. In the event that diagnostic uncertainty persists, it is recommended to repeat the test within a time frame of 2 to 3 weeks. Conversely, a negative result does not rule out, but makes it less likely; repeated testing may also be indicated if doubts remain (29,31). Differential diagnoses included autoimmune encephalitis (e.g., anti-NMDAR), metabolic and toxic encephalopathies, infections (HIV, syphilis) and neoplastic processes. These were excluded through comprehensive evaluation, and the clinical course and imaging were inconsistent with Alzheimer's disease, corticobasal degeneration, or other tauopathies (7,32).

The present case report highlights the importance of early neurological assessment when behavioral or cognitive decline progresses rapidly, particularly when initial symptoms mimic psychiatric illness. Early recognition enabled appropriate supportive and palliative management while avoiding unnecessary or invasive interventions (14).

In the context of the current literature, the present case report reinforces the need for vigilance when evaluating rapidly progressive neurocognitive syndromes. Despite diagnostic advances, establishing a definitive diagnosis without neuropathological confirmation remains challenging. Emerging biomarkers, such as the real-time quaking-induced conversion (RT-QuIC) assay, tau proteins and neurofilament light chain show promise in complementing clinical and imaging criteria, potentially reducing reliance on postmortem confirmation (31,33-35).

The case presented herein is distinctive for its initial psychiatric misdiagnosis, rapid clinical decline and clear demonstration of classical sCJD markers, allowing a robust diagnosis based on combined clinical, imaging and laboratory evidence. Some limitations include the absence of neuropathological confirmation, which remains the gold standard for definitive diagnosis, and the single-patient design, which limits generalizability. Nevertheless, the combination of clinical, imaging and biomarker findings strongly supports a probable diagnosis of sCJD. Future research is required to focus on validating integrated biomarker panels and advanced neuroimaging techniques to improve early, non-invasive diagnosis and patient management.

## Data Availability

The data generated in the present study may be requested from the corresponding author.
